# Challenges and opportunities of a paperless baseline survey in Sri Lanka

**DOI:** 10.1186/1756-0500-7-452

**Published:** 2014-07-15

**Authors:** Duleeka W Knipe, Melissa Pearson, Rasmus Borgstrøm, Ravi Pieris, Manjula Weerasinghe, Chamil Priyadarshana, Michael Eddleston, David Gunnell, Chris Metcalfe, Flemming Konradsen

**Affiliations:** 1 School of Social and Community Medicine, University of Bristol, Bristol, UK; 2 South Asian Clinical Toxicology Research Collaboration (SACTRC), Faculty of Medicine, University of Peradeniya, Peradeniya, Sri Lanka; 3 Pharmacology, Toxicology & Therapeutics, University/BHF Centre for Cardiovascular Science University of Edinburgh, Edinburgh, UK; 4 Department of International Health, Immunology and Microbiology, Faculty of Health Sciences, University of Copenhagen, Copenhagen, Denmark; 5 Geographical Resource Analysis and Science (GRAS), C/O Department of Geosciences and Natural Resource Management, University of Copenhagen, Copenhagen, Denmark

**Keywords:** Sri Lanka, Computer, Handheld, Data collection, Randomised control trial, Epidemiology

## Abstract

**Background:**

Personal digital assistants (PDAs) have been shown to reduce costs associated with survey implementation and digitisation, and to improve data quality when compared to traditional paper based data collection. Few studies, however, have shared their experiences of the use of these devices in rural settings in Asia. This paper reports on our experiences of using a PDA device for data collection in Sri Lanka as part of a large cluster randomised control trial.

**Findings:**

We found that PDAs were useful for collecting data for a baseline survey of a large randomised control trial (54,000 households). We found that the PDA device and survey format was easy to use by inexperienced field staff, even though the survey was programmed in English. The device enabled the rapid digitisation of survey data, providing a good basis for continuous data quality assurance, supervision of staff and survey implementation. An unexpected advantage was the improved community opinion of the research project as a result of the device, because the use of the technology gave data collectors an elevated status amongst the community. In addition the global positioning system (GPS) functionality of the device allowed precise mapping of households, and hence distinct settlements to be identified as randomisation clusters. Future users should be mindful that to save costs the piloting should be completed before programming. In addition consideration of a local after-care service is important to avoid costs and time delays associated with sending devices back to overseas providers.

**Discussion:**

Since the start of this study, PDA devices have rapidly developed and are increasingly used. The use of PDA or similar devices for research is not without its problems; however we believe that the universal lessons learnt as part of this study are even more important for the effective utilisation of these rapidly developing technologies in resource poor settings.

## Background

Personal digital (or data) assistants (PDAs) are mobile hand-held devices which are increasingly used as a preferred method of data collection over traditional paper based approaches. Some of the benefits offered by PDAs have been detailed elsewhere [1-8]. These include the reduced costs of survey implementation and digitisation [5-7]; improved data security and quality [1-8]; reduced survey time [5]; and rapid availability of results [2-5,7,8]. The electronic data capture at point of collection is a noticeable advantage of PDAs for large population based studies. The innovators and early adopters of this technology have primarily been research groups from high income countries. A few studies in low and middle income countries have implemented this technology, but only studies based in Africa and the South Pacific islands have reported their experiences [2,4-7,9-14]. We believe that sharing experiences of the barriers and distinct benefits of this technology will help future users to be better informed and allow for the swifter adoption of these and similar technologies. The aim of the short report is to share our insights and experiences of using PDAs for field data collection in a rural Asian context as part of a large randomized control trial.

### Setting

The project is based in the North Central Province of Sri Lanka, in an agricultural region of the Anuradhapura district. This work is part of an on-going trial entitled “A community-based cluster randomised trial of safe storage to reduce pesticide self-poisoning in rural Sri Lanka” [15], which has been designed to evaluate the effectiveness of the introduction of household pesticide storage devices in reducing the incidence of fatal and non-fatal self-harm. The trial started recruiting households on 31 December 2010. Ethics approval was granted from the University of Peradeniya, Sri Lanka in March 2008, with amendments in January and July 2011. The Provincial Department of Health Services and national Ministry of Health have given their support to the study. The trial is registered on ClinicalTrials.gov ref: NCT01146496 (http://www.clinicaltrials.gov/ct2/show/NCT1146496). The design of the study required a baseline survey to be completed on all households (approximately 54,000 households) within the study area and for households to be revisited during the follow-up period. All households are approached and given a brief introduction and the householders’ invited to verbally consent to participation. Consent can only be given by an adult member of the household (min. 18 years of age). In order to avoid long delays in data entry and follow-up, a PDA device with an inbuilt global positioning system (GPS) was utilised.

#### *The personal digital assistant (PDA)*

Our three main considerations when selecting a PDA device for this survey were: unit cost, robustness and usability. The study area experiences very high temperatures and humidity. The nature of the survey meant that the device had to withstand this climate and high levels of dust, very bright sunlight and increased likelihood of damage caused by accidental dropping and transport of the device. At the time of selecting a device the Trimble Juno SB handheld PDA unit was considered to be cheapest per unit, highly robust and most usable because of the screen size and visibility under sunlight. In addition to the considerations already described, this device was chosen for its integrated GPS functionality, and its long-life battery (internal 4600 mAh lithium ion battery – 8 hours). The device also has a short recharge time of only approximately four hours.

**Figure 1:**
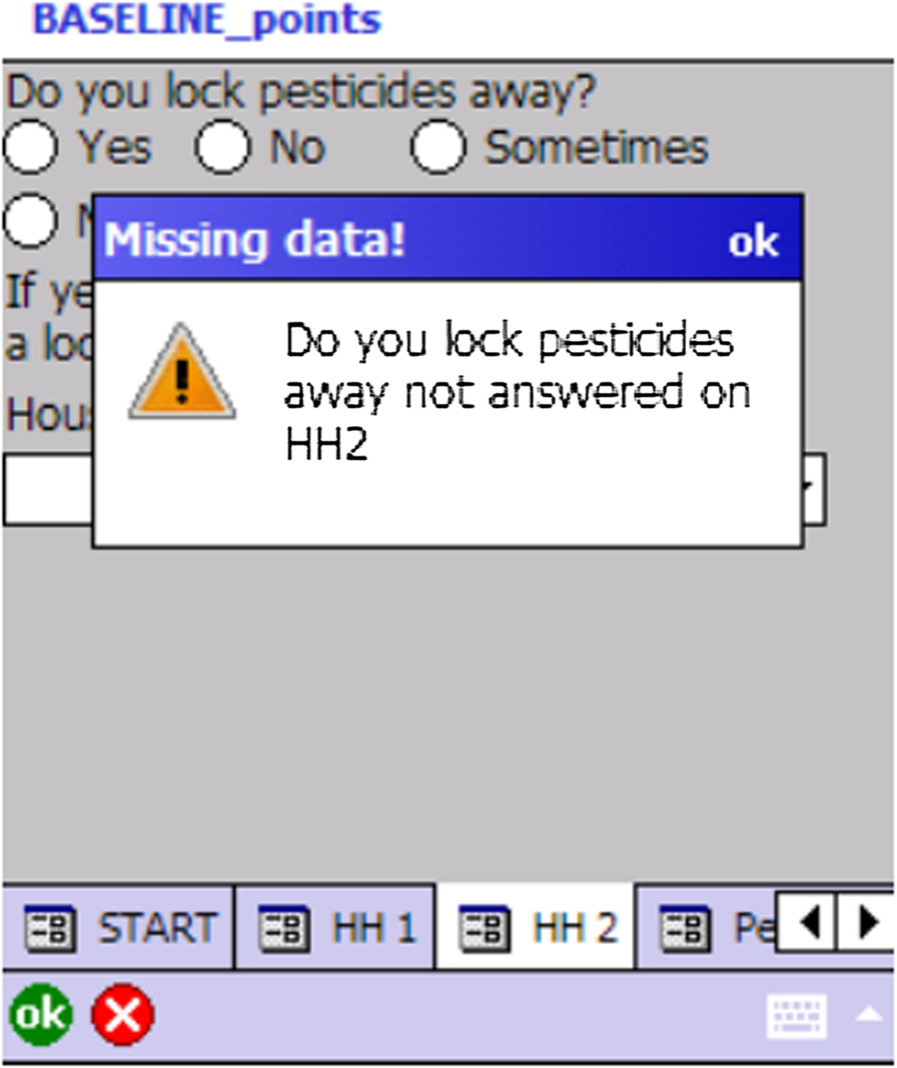
**Screenshot of a skip error warning on the PDA.** The figure illustrates an example of how skip errors are avoided. Certain questions are mandatory and data collectors must enter these data. For example every household entered into the device must have a response recorded as to whether pesticides are locked away, either “yes” or “no”. If the data collector misses this field, a warning message is displayed, and the survey cannot progress.

A total of 22 devices were purchased in three batches from two providers (Denmark and United Kingdom). The PDA was protected by an outer box and screen cover, had a screen size of 8.9 cm (diagonal length) and weighed 0.23 kg (with battery). The device was loaded with Microsoft Windows Mobile and the data collection pro-forma was designed with scripts within ArcPad 9.0 software. This enabled the survey questionnaire to be saved in formats compatible with state-of-the-art statistical and GIS (Geographic Information Systems) software for further post-processing and mapping of the survey locations. The survey was designed to appear on the device in English as the ArcPad software does not support the language script of the local population’s Sinhala language. Additional file 1 shows a data collector using the device for a household survey.Answers to the survey questions along with GPS co-ordinates were recorded for all consenting households. The data were entered using dropdown boxes, check boxes, and text fields. In order to maintain data quality the survey was programmed to use pre-programmed responses, avoid skip errors (see Figure 1) and included validation rules to ensure data entry for compulsory fields. Data were stored on the device on a micro secure digital (SD) card. Data were downloaded daily by field supervisors and backed up in three geographically separate locations and managed using Microsoft Access.

After data were downloaded, the GPS coordinates of surveyed households were plotted onto Google Earth. With the mapping of data collection points and local knowledge, the field team were able to mark village boundaries using both Google Earth and ArcGIS. This information was not available from routine sources as over time the administrative and village limits in the study area have changed, with poorly defined boundaries.

### Methods for gathering feedback

In order to provide a range of views of the different challenges and benefits faced when using this device, we included both author reflections and collected insights from field staff in Sri Lanka. The field staff were interviewed by the first author (DWK) in Sinhala following their verbal consent. These interviews were conducted after approximately half of the data collection was completed (26,500 households). A mixture of focus group discussions and interviews were employed to collect feedback from the 2 research managers, 3 field supervisors and 14 data collectors. The issues covered in the interviews included: training, user and respondent acceptability of the device and device performance. The participants were encouraged to discuss points outside of these general areas. Notes were taken during the interview and once all the interviews were completed, notes were reviewed to identify common themes.

## Findings

The feedback from the team and author reflections are presented in relation to broad topic areas.

### Device programming

Programming the initial/pilot data collection pro forma took approximately 40 hours and was undertaken at an early stage before the piloting of the questionnaire was complete. This resulted in the need for an additional 25 hours of re-programming. The additional costs for re-programming were unanticipated, and thus influenced the number of changes possible to implement and introduced delays. The authors felt that these costs could have been reduced if more extensive piloting of a hard copy of the questionnaire had been completed prior to initial programming.

The programming of the survey was done overseas. Sri Lanka has a growing number of people who have studied and become skilled in software designing. It may have been possible that one of these software designers could have programmed the device. We were, however, not aware of this during the initial programming of the device and therefore we did not investigate this further.

### Training and support for data collectors

Our initial expectations were that substantial training and support would be needed for data collectors to use the device programmed in English. The training included a residential classroom component followed by in-field training. All data collectors had completed high-school, had varying levels of English language skills and none had previous experience of using a hand held computer device for data collection. Data collectors were trained on the paper version of the questionnaire first and then introduced to the PDA device. It took roughly 2 days to be able to enter data effectively into the device, and survey time reduced rapidly in the first few weeks as data collectors became more familiar with the PDA.

Data collectors reported they were able to understand and navigate the questionnaire well following the training. Data collectors had available a Sinhala paper version of the questionnaire during the survey which gave definitions of survey responses, if needed. We also provided data collectors with a Sinhala script on the exact wording of the questions (paper format) for the survey. However, data collectors reported that after the initial training period they did not refer to the Sinhala paper copy. One manager interviewed felt that as the limited screen size meant that questions appearing on the device were very concise, with little explanation/prompts being available, there was potential for confusion and a need for retraining at regular intervals. Regular shadowing of data collectors ensured quick identification of any deviations from the survey script. If deviations were noted, these were corrected either on an individual basis or as a whole during team meetings.

### Supervision

**Figure 2:**
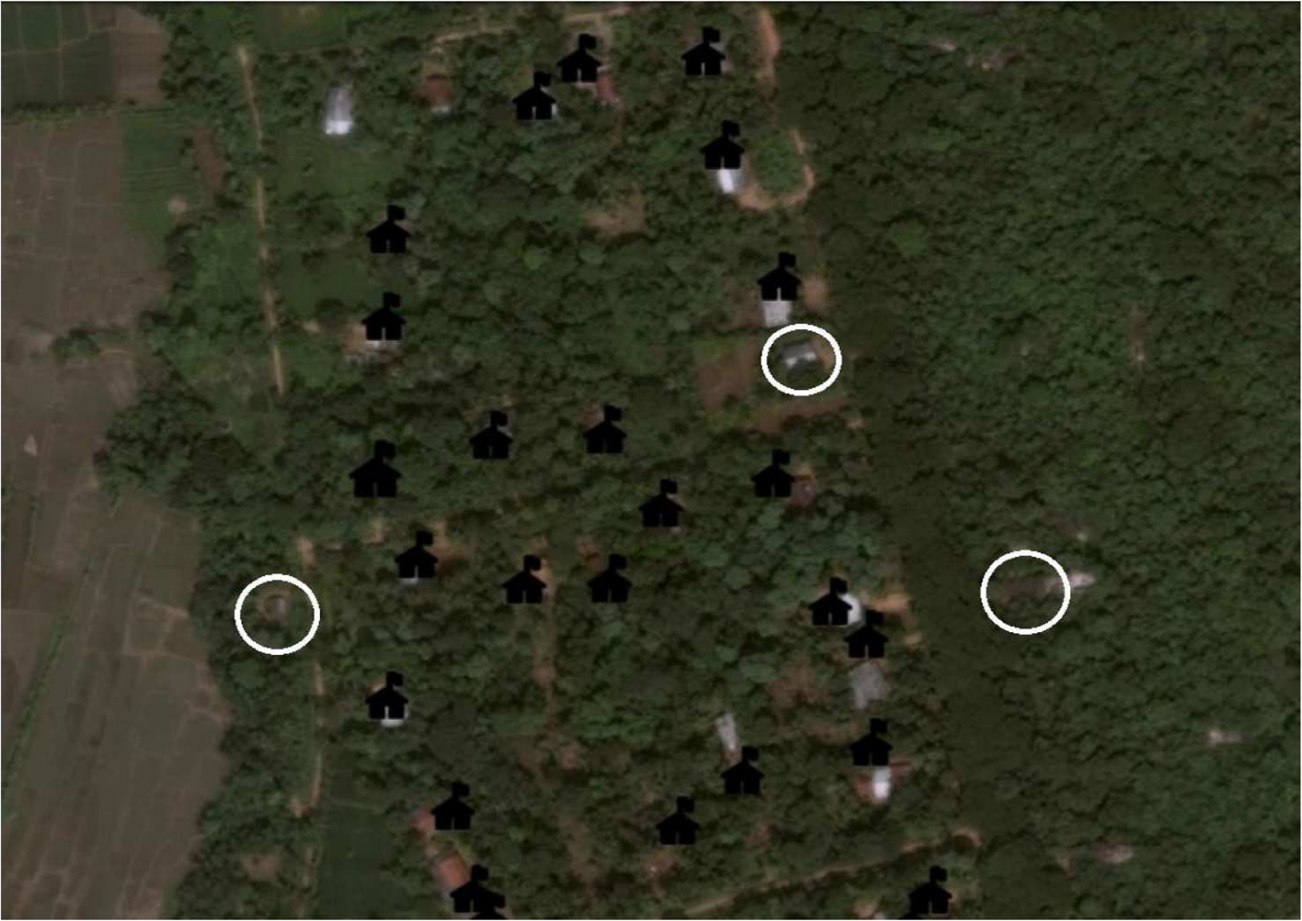
**Screenshot of identification of missing households.** The figure illustrates how by using Google Earth (Earth data: Google, DigitalGlobe) and the GPS data, the progress of data collection can be monitored. The locations of completed household surveys are marked using house icons. The households circled in white are households which were missed. The supervisors were able to direct data collectors by using these maps, to ensure that all households were approached. *Source*: “Missed households.” 8°08’28.27” N and 80°19’35.42” E. Google Earth. April 4, 2011. May 15, 2013.

Field supervisors reported that they found the automatic recording of GPS coordinates very helpful. This was especially so when they had to work from an office location, as the data collector could call in their GPS location, and the supervisor could guide them on their next route via telephone with the aid of Google Earth (Google©2013). In addition daily and weekly reports were generated automatically to allow the field supervisors to identify missed households (see Figure 2) and monitor data quality. These weekly summary quality reports also ensured that missing data (e.g. gender of individuals); and incorrect data (e.g. erroneous village name), could be identified and corrected quickly, helping to avoid systematic errors.

Managers and supervisors believed that they needed extensive training on the use of the device before its introduction to data collectors to allow for proper management of data collectors. The supervising staff perceived that during the initial stages the knowledge gap resulted in management difficulties. In a culture where it is very important for managers to be more knowledgeable than those they supervise, inabilities to solve technical problems with the device resulted in challenging management situations. As the managers grew in confidence and practical experience with the device, it improved their capacity to effectively supervise data collection.

### Survey implementation

The field supervisors reported that the battery life was sufficient for a full day’s survey (8 hours), provided the battery was fully charged. They did report, however, that on occasion due to power outages overnight a full day’s coverage was not possible the following day, especially if the backup battery store was also affected.

Data collectors believed that survey time was greatly reduced because of the ease of entering long Sri Lankan names; the data collector would only have to type in the long family name once and for subsequent members the family name could be auto-filled. Conversely whilst managers and supervisors acknowledged the benefit of the auto-fill function, they highlighted the risk that errors would be recorded several times before being noticed, if at all. The data collectors also reported that the screen visibility was poor when the data had to be collected outside without shelter. However they reported that the backlight was a useful feature when surveying in the evenings from households with no electricity. They also reported that the handheld aspect of the device allowed for surveying to be carried out whilst standing. This was particularly useful when surveying in shops and avoided embarrassment in poorer households where seating facilities were not available.

### Device acceptability

Initial concerns that respondents would be unwilling to give information due to suspicion about the device were unfounded. One manager reported that due to the wide availability of mobile phones, even in rural settings, the use of these “small computers” was readily accepted. Data collectors reported that the device encouraged interest in the survey, as it gave the project a higher status amongst villagers. Conversely there were also reports from data collectors that some villagers were concerned that they were being recorded/photographed/videoed, these concerns were easily overcome by a careful explanation of the purpose of the device. The data collectors found that households with security force employees were curious of the GPS functionally and asked additional questions. This is a problem that is likely to be specific to countries experiencing or having recently experienced political and/or civil unrest. Until recently (2009), Sri Lanka had suffered from a long civil war, and as a result any recording devices were generally viewed with suspicion. The data collectors felt they were able to provide reassurance in response to such concerns because of the research training they received.

### GPS functionality

Supervisors and data collectors also reported problems with recording a proper location (“fixing”), when the GPS signal was weak due to interfering objects (heavy cloud cover, houses, trees etc.) or bad GPS satellite constellations. It was possible to proceed without the device “fixing”, but if the survey was completed before the correct location was marked, then incorrect information would be recorded. To avoid this, a protocol was developed to wait until the device recorded a proper location before proceeding. Data collectors reported that it could take up to 15 minutes to get a proper location and at times this affected the rapport with household members, especially if they were disturbed at a busy time.

### After-care provision

Four of the twenty-two devices experienced hardware malfunctions during 78 weeks of data collection. No local after-care services were available; therefore devices had to be sent back to the overseas providers, with turnaround times lasting several months. The project management team identified this as adding significant cost and time delays, and required the field team to purchase additional backup devices.

### Data management

Given the sample size (54,000 households) and pace of data becoming available, it was essential to have database and data management process in place. The authors perceived that the device helped to secure data quality and ensure that no data was lost as result of paper questionnaires being mislaid or damaged in a field setting. These benefits would, however, be lost if stringent data management procedures are not in place, especially in terms of database management.

The ArcPad software that we used to create the survey on the device created a database file (.dbf) which we were able to import directly into Microsoft Access. We chose to use Microsoft Access as a database platform because of its user-friendliness. The post-survey databases were designed by one of the non-local authors of this manuscript, who was based in the study area at the time of the survey. The database system designed allowed for automatic uploads of the survey data from the survey device by field workers who were unskilled in Microsoft Access. Once the data was uploaded into the post-survey Access databases, the field workers were able to quickly generate automated quality and basic statistical reports for field use. Whilst we did have difficulty finding a suitably qualified person in our remote study area to do any more advanced analysis on site, the field team were able to extract and send the data offsite if any additional analysis was required.

### Added benefits to the trial

**Figure 3:**
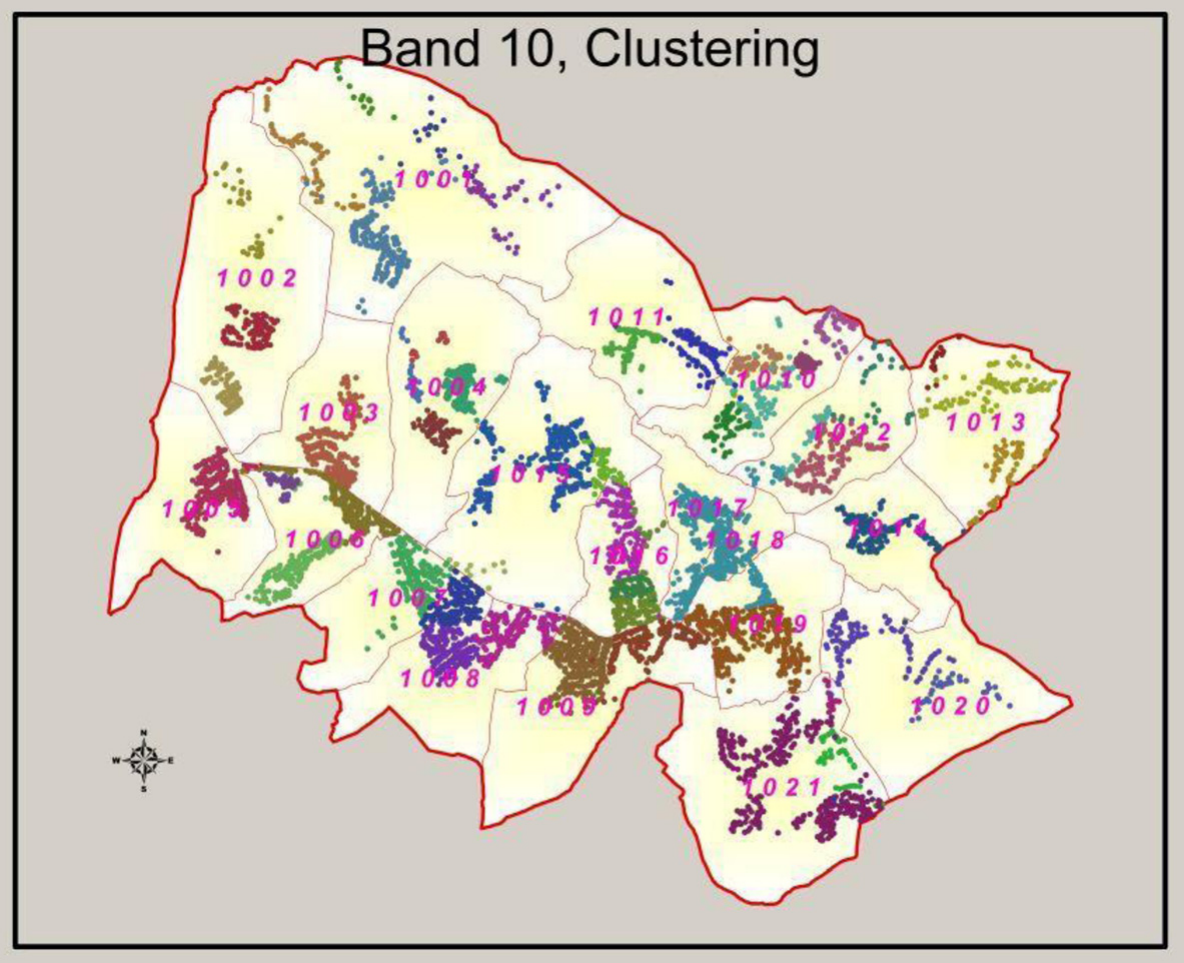
**Identification of cluster boundaries for randomisation.** The figure illustrates the finalised cluster boundaries used for a particular band within the study area. GPS points, local knowledge and GIS were used to identify approximate clusters. This was achieved by plotting all surveyed household within a certain area onto Google Earth. Using this as a tool alongside local knowledge, we would roughly draw cluster boundaries using the polygon drawing functionality in Google Earth. These rough boundaries were then translated into finalised cluster boundaries using ArcGIS.

For cluster randomised trials and other surveys where it is important to investigate area effects, it is essential to define geographic clusters to minimise the risk of contamination. Contamination can occur when individuals in the control arm of the trial observe or are otherwise prompted to adopt the study intervention. The project was designed using administrative boundaries to define clusters. These boundaries, however, often had closely neighbouring households on either side, with a risk of contamination if those adjacent administrative areas were allocated to different arms of the study (see [15] for further discussion). The use of GPS points, along with local knowledge, allowed for acceptable cluster identification post-survey (Figure 3).

An added advantage reported by managers and field supervisors was that as the trial requires revisits to the field, the recording of the GPS location of households in remote areas proved invaluable for relocating these houses for follow-up.

## Conclusions

PDA devices provide several benefits when used in resource poor settings, but our experiences highlight important considerations for future users. An important lesson learnt was that adequate piloting (hardcopy) of the baseline survey before programming is needed to ensure that the costs with reprogramming are kept to a minimum. Contrary to our reservations that training and acceptability of a PDA device programmed in English for inexperienced staff would be difficult, we found that the training of data collectors in the use of the PDA did not take long, and the device was readily accepted. Managers, however, felt they needed more extensive training to overcome technical difficulties in order to manage effectively. A significant advantage of the PDA was the digitisation of data at point of entry, as this allowed for rapid statistical updates on coverage and data quality. The inbuilt GPS functionality also allowed for prompt mapping of progress using Google Earth, this in turn made logistical planning, supervision and boundary definition much easier in a large survey. The digitisation also ensured that the time lag between data collection and analysis was minimised. We found that the device was readily accepted in the community and provided an additional tool for attracting interest to the survey. There were, however, some problems with the devices not “fixing” which resulted in difficulty maintaining rapport with household members. An important consideration for potential users is to ensure that an alternative protocol for data collection is in place. Future users should also consider the importance of selecting a device which can be repaired locally, as sending devices abroad for repairs is expensive.

PDA devices provide an invaluable tool for research, as theyreduce the resources needed for digitisation, helps improve data quality and security. Since the start of this study, PDA devices have rapidly developed with the introduction of smartphones and tablets. These introductions coupled with low cost applications, are likely to rapidly boost the use of handheld devices. With this expected advance in both handheld device technologies and the accompanying increasing use of these devices, we believe that the universal lessons learnt as part of this study are even more important for the effective utilisation of these rapidly developing technologies.

## Abbreviations

PDA: Personal digital assistant; GPS: Global positioning system; GIS: Geographic Information Systems; SD: Secure digital.

## Competing interests

Authors declare no competing interests.

## Authors’ contributions

DWK, MP and FK designed the interviews/focus group discussion. DWK conducted and analysed the interviews/focus group discussions and wrote the first version of the manuscript. MP is responsible for the day to day management of the trial. RP, MW and CP lead the field work teams and participated in the interviews/focus group discussions. RB programmed the PDA devices and contributed text to the manuscript. ME, FK, DJG, CM, RP, and MW contributed to the original proposal for the trial, study design, and study progress. All authors reviewed the manuscript and approved the final version.

## Supplementary Material

Additional file 1 Data collection using the Juno Trimble PDA. Image of PDA device being used for household data collection. Click here for file
