# Supplementary figures and images for: Challenges and opportunities of a paperless baseline survey in Sri Lanka
Source: BMC Res Notes. 2014 Jul 15;7:452. doi: 10.1186/1756-0500-7-452 (PMC4118630; doi:10.1186/1756-0500-7-452)

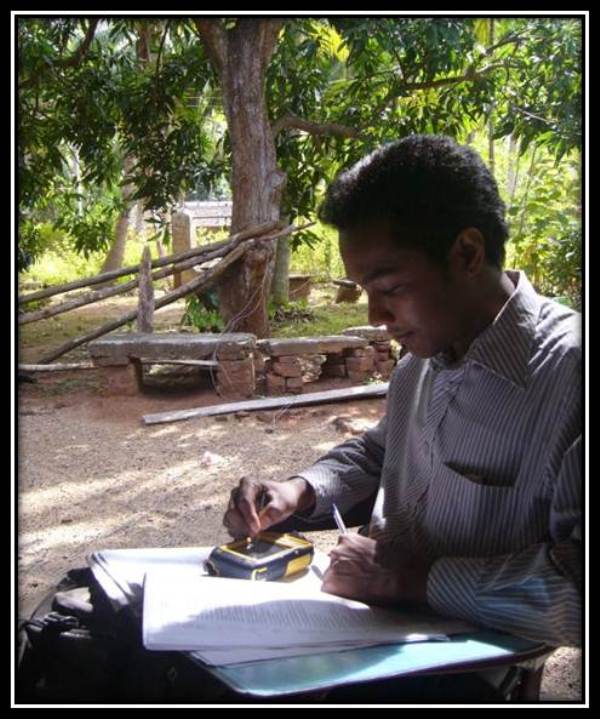

Supplement: Additional file 1 — Data collection using the Juno Trimble PDA. Image of PDA device being used for household data collection. [file 1756-0500-7-452-S1.jpeg]
